# Surgical Treatment Outcomes of Tetralogy of Fallot: Experience of the Cardiovascular Department of the Mohammed VI University Hospital in Marrakech

**DOI:** 10.7759/cureus.24773

**Published:** 2022-05-06

**Authors:** Ahmed Salah Tandia, Marwan El Qady, Saloua El Karimi, Driss Boumzebra

**Affiliations:** 1 Department of Cardiology and Vascular Diseases, Mohammed VI University Hospital, Marrakech, MAR; 2 Department of Cardiovascular Surgery, Mohammed VI University Hospital, Marrakech, MAR

**Keywords:** cyanotic congenital heart disease, interventricular communication, residual pulmonary stenosis, pulmonary insufficiency, tetralogy of fallot

## Abstract

Background

Tetralogy of Fallot (TOF) is the most common cyanotic congenital heart disease. The surgical approach to TOF continues to evolve, with many centers now favoring early repair for TOF. This study aimed to determine the prevalence of postsurgical complications such as pulmonary insufficiency, residual pulmonary stenosis, and interventricular communication.

Methodology

We conducted a cross-sectional, observational study with a descriptive and analytic focus involving 61 patients who were operated on for TOF.

Results

In total, 39 (63.9%) patients had a regular TOF and 22 (36.1%) had an irregular TOF. In our study, 32 (56.14%) patients had a pulmonary insufficiency, of whom 20 had a minimal pulmonary insufficiency (62.5% of pulmonary insufficiency cases), and 79.4% of patients with right ventricular outflow tract enlargement had pulmonary insufficiency (p < 0.005). Among patients who did not have a right ventricular outflow tract enlargement, three cases of pulmonary insufficiency were identified with a prevalence of 16.6%. Six (10.52%) patients had residual pulmonary stenosis. In addition, two (3.2%) cases of minimal residual ventricular septal defects were identified in this study.

Conclusions

Postoperative complications of TOF treatment are frequent and require medical care throughout the lifetime of patients.

## Introduction

Tetralogy of Fallot (TOF) is the most common cyanotic congenital heart disease in childhood accounting for 10% of congenital heart diseases. It is associated with four anomalies, namely, a ventricular septal defect, stenosis of the pulmonary pathway (infundibular, valvular), the aorta straddling the ventricular septal, and hypertrophy of the right ventricle. Heart disease is considered to be regular in the absence of the following abnormalities: an additional ventricular septal defect, coronary anomalies, an anomaly of the pulmonary arterial tree (in number, size, or origin), and irregular in the presence of at least one of these abnormalities. The treatment consists mainly of atriotomy under the extracorporeal circulation of a ventricular septal defect closure, resection of the pulmonary tract stenosis often with the placement of an infundibular enlargement patch, and correction of associated malformations. Sometimes before this complete cure, it may be necessary to first place a systemic pulmonary shunt to help postpone surgery because of conditions related to the patient such as low weight or to promote maturation of the pulmonary arterial tree. The complete cure, although often successful, is not free of complications, such as pulmonary insufficiency, pulmonary stenosis, or residual ventricular septal defect, which, depending on their severity, constitute major prognostic factors.

## Materials and methods

This is a cross-sectional study based on the postoperative profiles of 61 patients operated on for TOF from 2014 to 2019 in the Department of Cardiovascular Surgery, Mohammed VI University Hospital, Marrakech. This study was approved by the Faculty of Medicine and Pharmacy of Marrakech, a university linked to the University Hospital of Marrakech (020/2017). In this study, we investigated the preoperative profiles of these patients, as well as the surgical approach performed. All our patients were operated on by our cardiovascular surgery team and benefited from echocardiography performed between 6 and 12 months after the surgery. The echocardiograms were performed by the medical team that treats congenital heart disease at the Mohammed VI University Hospital in Marrakech. We included all patients who underwent a complete TOF cure. The objective of this study was to search for and evaluate pulmonary insufficiency, residual pulmonary stenosis, and interventricular communication.

## Results

**Figure 1:**
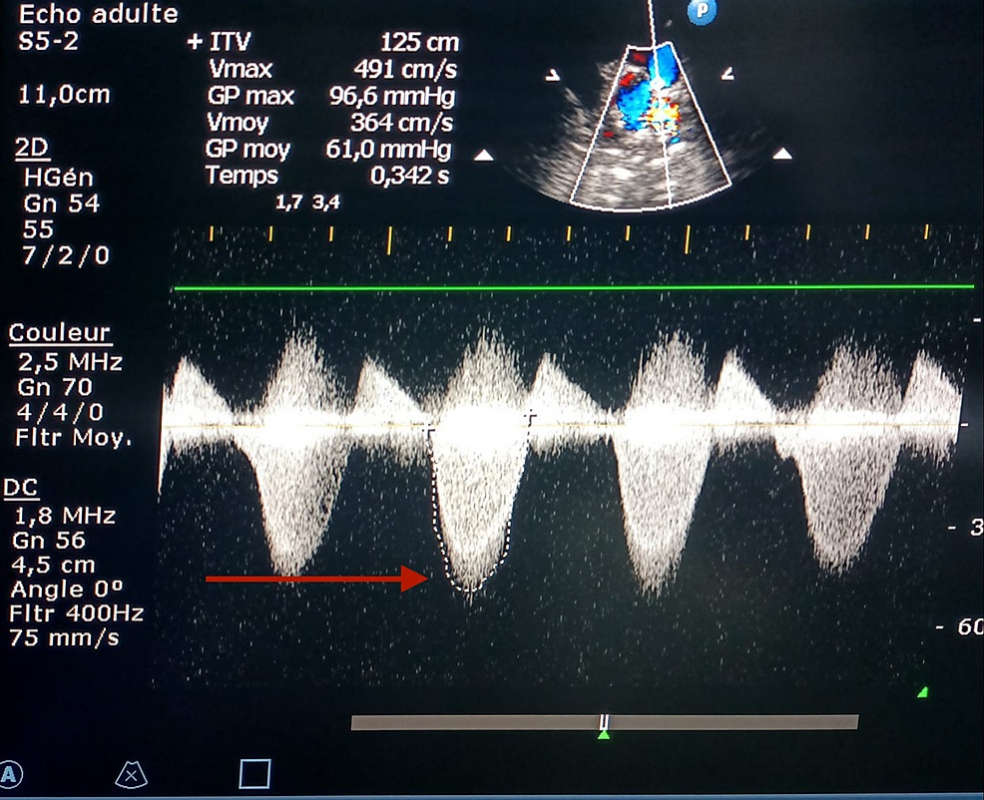
Pulmonary stenosis before surgery.

**Figure 2:**
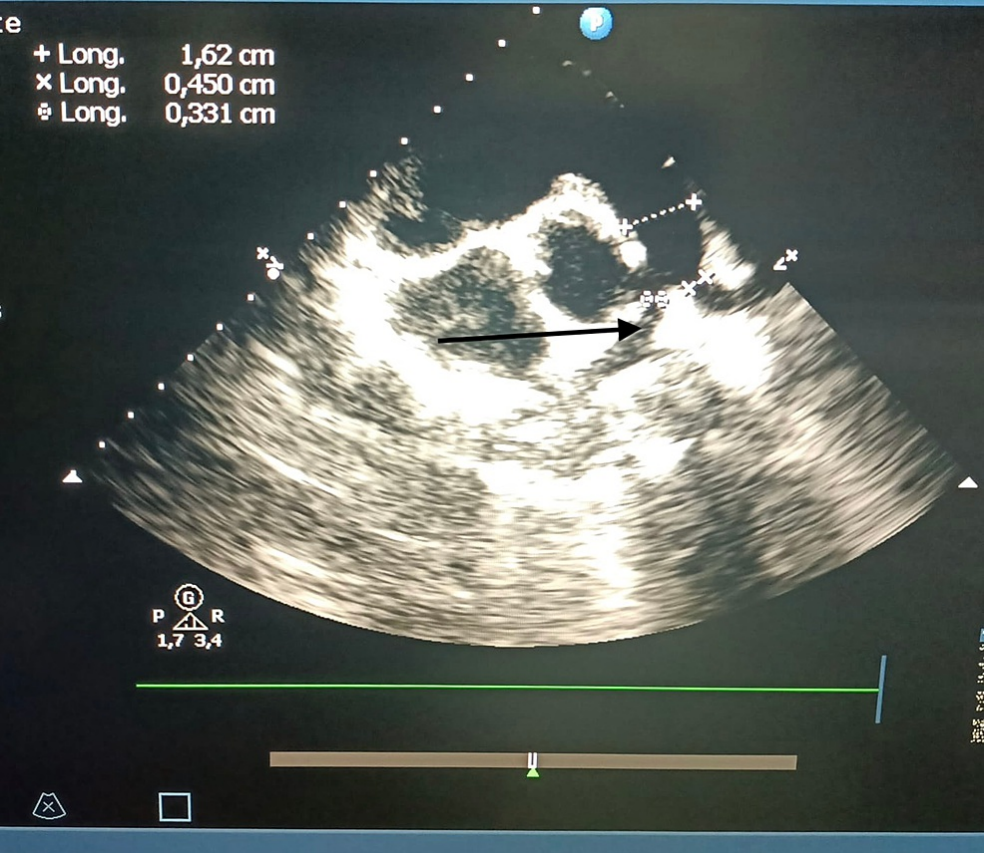
Pulmonary artery hypoplasia before surgery.

According to the presurgical profile of the patients in our series, 39 (63.9%) patients had a regular TOF type (Figure 1), and 22 (36.1%) patients had an irregular TOF type (Figure 2). The malformations associated with TOF in our series were as follows: 13 (21.3%) patients had an associated atrial septal defect, five (8.19%) patients had an associated persistent ductus arteriosus, and one (1.6%) patient had an associated coronary anomaly. Concerning pulmonary outlet stenosis, 11 (18%) patients had an infundibular stenosis, 15 (24.6%) patients had a severe infundibular stenosis, and 22 (36.1%) patients had a severe infundibular and associated valvular stenosis. One (1.6%) patient had pulmonary artery stenosis at the bifurcation (Table 1).

**Table 1:** Presurgical profile of the patients. TOF: tetralogy of Fallot; ASD: atrial septal defect; PDA: persistent ductus arteriosus; CA: coronary anomaly; PASB: pulmonary artery stenosis at the bifurcation

Presurgical profile		Percentage %
Type of TOF	Regular type	63.9
	Irregular type	36.1
Malformations associated	ASD	21.3
	PDA	8.19
	CA	1.6
	PASB	1.6

In our series, 57 (93.4%) patients benefited from closure of the ventricular septal defect by pericardial patch and four (6.6%) patients benefited from a Dacron patch. In our series, all patients underwent infundibular resection via the right atrial approach. In cases where infundibulectomy was deemed unsatisfactory, an additional approach via ventriculotomy was performed.

In total, 34 (70.5%) patients underwent right ventricular outflow tract enlargement by patch. Infundibular enlargement alone was performed in four (6.55%) patients. Right ventricular infundibulum associated with pulmonary artery annulus enlargement was performed in 30 (49.18%) patients. In total, 27 (44.32%) patients did not receive the right outlet enlargement. Twenty-one (34.4%) patients had pulmonary valve commissurotomy (Table 2).

**Table 2:** Surgical modalities. VSD: ventricular septal defect; RVOT: right ventricular outflow tract; PAA: pulmonary artery annulus

Surgical modality			Percentage
Closure of VSD	Pericardial patch		93.4
	Dacron patch		6.6
RVOT enlargement	Yes	Infundibular enlargement alone	6.5
		RVOT + PAA enlargement	49.18
	No		44.32
Pulmonary valve commissurotomy			34.4

**Figure 3:**
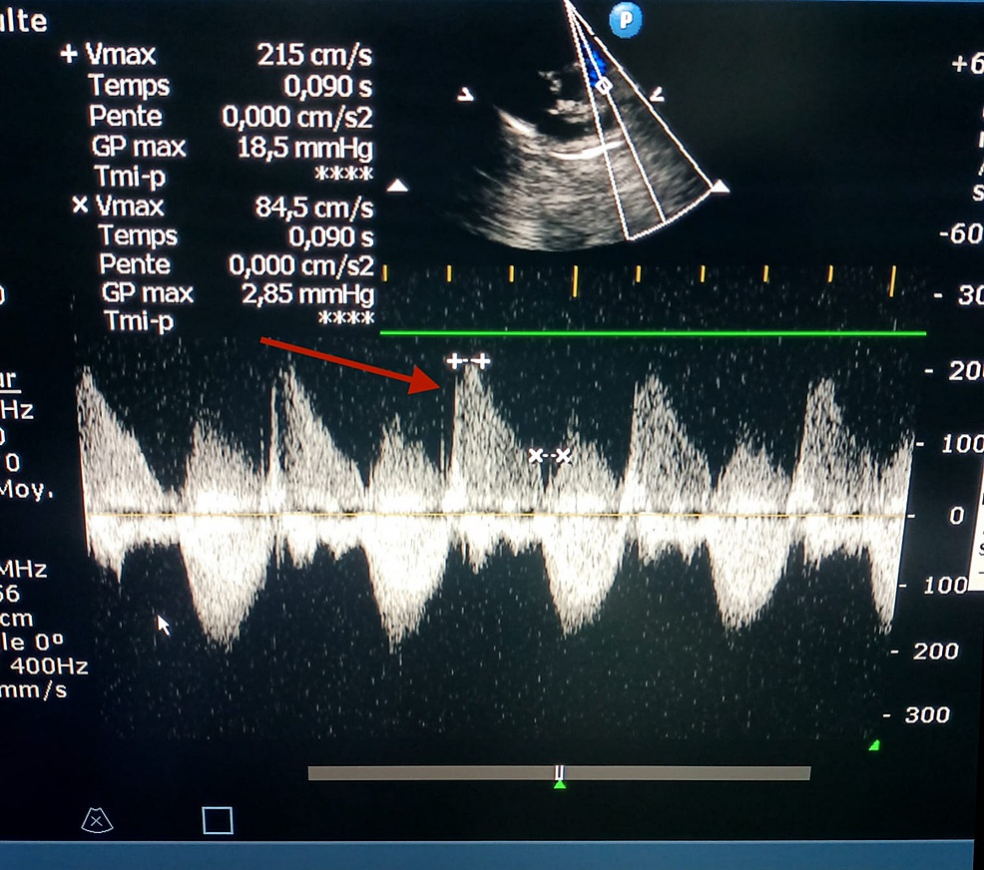
Pulmonary insufficiency after surgery.

In our study, 32 (56.14%) patients had a pulmonary insufficiency (Figure 3), of whom 20 patients had a minimal pulmonary insufficiency (62.5% of pulmonary insufficiency cases), nine patients had a moderate pulmonary insufficiency (28.12% of pulmonary insufficiency cases), and three patients had a severe pulmonary insufficiency (9.37% of cases). No pulmonary insufficiency was observed in 25 (43.85%) patients. In a statistical analysis correlating pulmonary insufficiency and right ventricular outflow tract enlargement by patch (pericardial or dacron), 79.4% of patients with right ventricular outflow tract enlargement had pulmonary insufficiency (p < 0.005). In patients who did not have a right and right ventricular outflow tract enlargement, three cases of pulmonary insufficiency were identified with a prevalence of 16.6%. In 67% of cases with severe pulmonary insufficiency, infundibular and pulmonary annulus enlargement combined with commissurotomy was performed, and in 33% of cases, infundibular and pulmonary annulus enlargement was performed.

**Figure 4:**
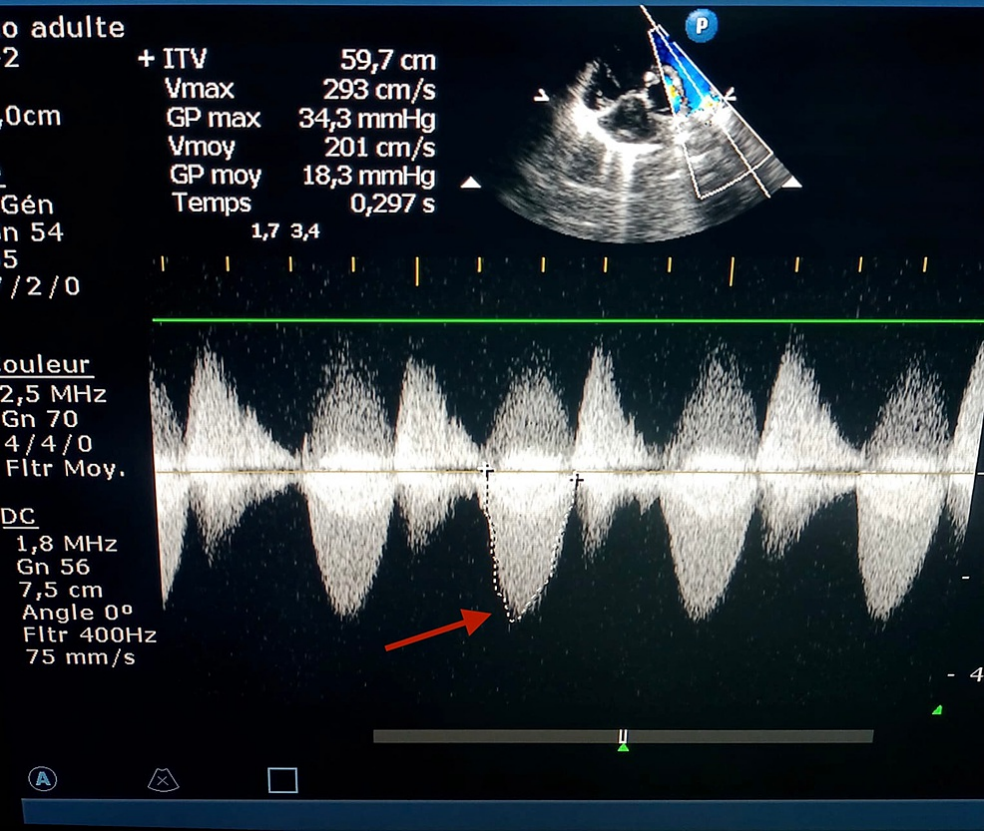
Residual pulmonary stenosis after surgery.

In our series, six (10.52%) patients had residual pulmonary stenosis (Figure 4). Of these, stenosis was minimal in four (66.66% of stenosis cases) patients and moderate in two (3.33% of stenosis cases) patients. The right ventricular outflow tract was free in 51 (89.5% of the total series) patients.

In our study, two cases of minimal residual ventricular septal defect were identified for a prevalence of 3.2% (Table 3).

**Table 3:** Control results.

Complications	Global percentage	Gravity	Relative percentage
Pulmonary insufficiency (PI)	56.4	Minimal	62.5% of PI cases
		Moderate	28.12% of PI cases
		Severe	9.37% of PI cases
Residual pulmonary stenosis (RPS)	10.52	Minimal	66.6% of RPS cases
		Moderate	33.3% of RPS cases
Residual ventricular septal defect (RVSD)	3.2	Minimal	100% of RVSD cases

## Discussion

A complete surgical cure provides a 40-year survival rate of at least 90% [1]. However, even if the survival rate is good, the complications related to the surgery determine the medium and long-term prognosis of patients.

The right ventricle, after surgical repair, has several potential developments, most often in adults, for which guidelines recommend regular follow-up (at a minimum of once a year) in a specialized center [2]. The purpose of this regular follow-up is to detect lesions requiring re-intervention on time before irreversible complications occur. Doppler echocardiography remains a valuable technique in postoperative assessment. It allows the diagnosis of pulmonary insufficiency, residual pulmonary stenosis by measuring the gradient across the pulmonary artery, and residual ventricular septal defect, as well as the quantification of the hemodynamic consequences [3].

Pulmonary valve insufficiency is very frequent and noted in about 60% of the cases according to Friedli [4]. The placement of a transannular patch during the operation, which leaves a part of the orifice unguarded, is the main factor responsible for this regurgitation. Regurgitation is mild in the majority of cases and is reported to be well tolerated for many years. In about 10% of cases [4], valve insufficiency is moderate to severe.

In our series, the frequency of pulmonary insufficiency was 56.14%, of which minimal pulmonary insufficiency was seen in 62.5% of cases. As described in the literature, we noted a close correlation between pulmonary insufficiency and right ventricular outflow tract enlargement with a frequency of 90.6% in patients benefiting from a right ventricular outflow tract enlargement against 3.2% in those not benefiting from a right ventricular outflow tract enlargement (p < 0.005).

Residual pulmonary stenosis must be investigated because it leads to a pressure overload on the right ventricular, already submitted to volume overload. This stenosis can be located at different levels, namely, at the infundibulum, pulmonary valve, pulmonary truncus, or pulmonary branches [5]. In our series, the frequency of residual pulmonary stenosis represents 10.52% of the cases, and among those, the stenosis was minimal in 66.66% of cases and moderate in 33.33% of cases. This frequency has also been reported in the series by Vaujois et al. who reported 12.5% of cases [6]. However, in our study, as well as in the literature, no correlation has been established between the architecture of the pulmonary tract preoperatively (type of stenosis) and the residual pulmonary stenosis. This could be explained by an attitude of surgeons to prefer a right ventricular outflow tract enlargement than to risk the persistence of a gradient on the right ventricular outflow tract. A residual ventricular septal defect is not very rare but usually minimal and without hemodynamic impact. Moderate-to-large shunted ventricular septal defects are not well tolerated and require re-intervention [7]. Minimal residual ventricular septal defects were identified in 3.2% of cases in our series. This proportion is comparable to that reported in the majority of foreign series [8]. Indeed, most authors have reported a frequency varying between 6% and 16.2%. In our study, no correlation was found between the frequency of residual ventricular septal defect and the consumables used during the closure (Dacron or bovine pericardium and porcine).

This study had some limitations. We recognize the limited number of patients included in the study. In the complications studied, we did not evaluate the dysfunction of the right ventricle given the low sensitivity of the cardiac echo-Doppler. MRI remains the gold standard for the evaluation of right ventricular function but is difficult to access.

## Conclusions

Sequelae and/or complications make medical control necessary throughout the life of surgery patients. They also remind us that surgical repair cannot restore completely normal anatomy. It should be noted that we must also give ourselves more possibilities, especially during the rigorous follow-up of these operated patients, and also for adequate management for the prevention and management of different complications. We must not forget that cardiac surgery has radically transformed the prognosis of this malformation and that a majority of operated patients can have a normal professional and family life.
